# Role of human papillomaviruses in carcinogenesis

**DOI:** 10.3332/ecancer.2015.526

**Published:** 2015-04-29

**Authors:** Raffaella Ghittoni, Rosita Accardi, Susanna Chiocca, Massimo Tommasino

**Affiliations:** 1 Department of Biological Sciences, Molecular and Computational Biology, University of Southern California, Los Angeles, CA 90089, USA; 2 Infections and Cancer Biology Group, International Agency for Research on Cancer, 150 cours Albert Thomas, 69008 Lyon, France; 3 IFOM-IEO Campus, Via Adamello 16, 20139 Milan, Italy

**Keywords:** papillomavirus, carcinogenesis, HPV vaccines

## Abstract

The human papillomavirus (HPV) family comprises more than 170 different types that preferentially infect the mucosa of the genitals, upper-respiratory tract, or the skin. The ‘high-risk HPV type’, a sub-group of mucosal HPVs, is the cause of approximately 5% of all human cancers, which corresponds to one-third of all virus-induced tumours. Within the high-risk group, HPV16 is the most oncogenic type, being responsible for approximatively 50% of all worldwide cervical cancers. Many studies suggest that, in addition to the high-risk mucosal HPV types, certain cutaneous HPVs also have a role in the development of non-melanoma skin cancer (NMSC).

Functional studies on the HPV early gene products showed that E6 and E7 play a key role in carcinogenesis. These two proteins use multiple mechanisms to evade host immune surveillance, allowing viral persistence, and to deregulate cell cycle and apoptosis control, thus facilitating the accumulation of DNA damage and ultimately cellular transformation.

The demonstration that high-risk HPV types are the etiological agents of cervical cancer allowed the implementation in the clinical routine of novel screening strategies for cervical lesions, as well as the development of a very efficient prophylactic vaccine. Because of these remarkable achievements, there is no doubt that in the coming decades we will witness a dramatic reduction of cervical cancer incidence worldwide.

## Members of the human papillomavirus family and their clinical implications

The human papillomaviruses (HPV) consist of a heterogeneous group of capsid-enclosed dsDNA viruses from the *Papillomaviridae* family that display a distinct tropism for mucosal or cutaneous squamous epithelia. Until now, more than 170 HPV types have been isolated and characterized [1].

Based on the homologous nucleotide sequence of the major capsid protein L1, a HPV phylogenetic tree has been generated that groups the different HPV types in genera [1, 2]. The alpha genus consists in approximately 30 HPV types that infect the mucosa of the genital tract as well as several cutaneous HPV types, e.g. HPV2 that is responsible for common skin warts. There are two groups of mucosal HPV types: low-risk HPVs (e.g. types 6 and 11), which are mainly linked to benign genital warts, and high-risk HPVs which are the etiological agents of cervical cancer and a subset of other human cancers (also see below) [3]. A large group of cutaneous HPV types forms the beta genus and are suspected to be involved, together with UV irradiation, with the development of non-melanoma skin cancer (NMSC) [4–6]. The other genera, gamma, mu and nu comprise cutaneous HPV types that are usually linked to the development of the cutaneous papillomas and warts.

While the involvement of HPV in causing benign warts was already known, the first evidence of association between human cancer and certain HPV types was proposed thirty years ago by zur Hausen and colleagues [7]. Subsequent epidemiological and biological studies confirmed the direct role of several mucosal HPV types in the development of cervical cancer and other epithelial tumors [8–10]. Worldwide epidemiological studies indicate that 18 different high-risk HPVs, namely 16, 18, 26, 31, 33, 35, 39, 45, 51, 52, 53, 56, 58, 59, 66, 68, 73, and 82, are associated with cervical cancer [9, 11]. HPV16 and HPV18 are the most carcinogenic types within this group, being responsible for approximately 50% and 20% of cervical cancer respectively [9, 12]. A subset of anal, penile, vulvar, vaginal, and oropharyngeal cancers have been attributed to the infection with high-risk HPVs [13, 14]. Notably, these cancers, in contrast to cervical cancer, appear to be mainly associated with HPV16, e.g. in more than 90% of the HPV-positive oropharyngeal cancers [15–17] (Table 1).

The first indication for the oncogenic potential of beta-HPV types came from their isolation in the skin of patients suffering from a rare genetic disorder called *Epidermodysplasia verruciformis* (EV) [18, 19]. EV patients have an impaired immune system and have high susceptibility to widespread and persistent HPV infection of the skin. As a consequence, they develop extensive verrucosis of confluent flat warts, which in approximately 30–60% of cases evolve into multifocal squamous cell carcinoma (SCC) in sun-exposed regions. Accordingly, organ transplant recipients under immunosuppression treatment have a 50–100-fold increased risk of developing NMSC, and their skin is highly positive for beta HPV types [20]. Through the use of more sensitive diagnostic assays, it is clear nowadays that beta HPV-type infection is highly frequent also in the skin of healthy individuals [21]. However, their involvement in the development of NMSC in the general population is still not entirely demonstrated.

Determination of beta-HPV viral load by quantitative PCR revealed that only a minority of skin cancer cells contained viral DNA [21, 22]. A plausible hypothesis is that the beta- HPV types play a role in the initial phase of carcinogenesis and are not required in the later stages for maintenance of the neoplastic phenotype. This situation differs from that established for mucosal high-risk HPV in cervical cancer, where the expression of viral oncogenes is constantly required during the entire carcinogenic process [7]. This may imply a need to consider a new scenario for the role of beta-HPV in NMSC pathogenesis, such as a ‘hit-and-run’ mechanism, although this by some extent may be regarded as controversial. Ultraviolet (UV) light is a well-known risk factor for skin cancer [23–25], inducing irreversible DNA damage. Thus, it is likely that beta-HPV types act as facilitators of accumulation of UV-induced DNA mutations, but are not required for the maintenance of cancer.

## Screening strategies for HPV-associated cervical diseases

In the last three decades, cervical cancer incidence has been constantly decreasing in high-resource countries, because of the introduction of national cervical cancer screening programmes. In contrast, in low-resource countries, because of the lack of screening programmes, cervical cancer still remains a serious health problem being the first or second cancer in women [26]. The conventional Pap smear or the liquid-based cytology methods are the most frequently used approaches for cervical cancer screening, and are based on the morphological analysis of cervical exfoliated cells. The performance of these screening methods is strongly dependent on staff training. In fact, in countries were Pap test is not properly implemented yet, such screening method may frequently lead to misdiagnosis [27, 28]. Independent studies have shown that HPV DNA detection, used as a primary screening, has a higher sensitivity and negative predictive value for the detection of pre-invasive disease than the conventional Pap smear or the liquid-based cytology methods [29–32]. HPV testing can detect approximately 50% more cervical intraepithelial neoplasia (CIN) than the Pap test [33]. In addition, a recent study in India demonstrated that the use of HPV DNA testing once in life reduces mortality from invasive cervical cancer to approximately 50% [34]. The negative predictive value for combined HPV and cytology testing is very high, which is of particular interest for the cost efficiency of a cervical cancer screening [31, 35, 36]. An HPV typing method more widely used in clinical and epidemiological studies is a non PCR (polymerase chain reaction)-based commercial liquid hybridisation assay Hybrid Capture 2 (HC2) (Digene) which can detect 13 high-risk HPV types (16, 18, 31, 33, 35, 39, 45, 51, 52, 56, 58, 59, and 68) and five low-risk HPV types (types 6, 11, 42, 43, and 44) [37]. However, this method can only detect the presence of HPV infection, without revealing the HPV type. Additional assays have been developed and commercialised that can identify the HPV types present in the cervical specimens, e.g. LINEAR ARRAY HPV Genotyping (Roche Molecular Diagnostics) and INNO-LIPA HPV genotyping (Innogenetics). A limitation of all HPV detection methods is their low positive predictive value, not being able to discriminate between HPV-positive infections with morphological alterations and those without [38]. Indeed HPV infections can be often found, especially in young women, however in a majority of the case those infections are not persistent and will be naturally cleared by the immune system without inducing cervical lesions [39].

Because of this constrain of the HPV detection assays, the interpretation of HPV-positive data require a particular attention and further analyses. It is now clear that additional markers need to be considered to improve the positive predictive value of the HPV detection assays. Many studies suggest that analysis of viral load [40, 41], HPV transcripts [42] and/or expression of specific host genes [43] will facilitate the identification of HPV infections associated with cervical lesions. However, additional studies are required to corroborate the initial findings and develop strategies that can be adopted in the routine clinical procedures.

Recently, over-expressed p16^INK4a^ (p16) has been described as a surrogate biomarker of HPV-induced cellular transformation [44]. A further example of a putative biomarker is reported in this issue. Specifically, in HPV-positive cervical cancer lesions there is a significant increase of the SUMO conjugating enzyme Ubc9 in CIN 2/3 cervical biopsies, compared to CIN 1 and to non-infected tissues (Mattoscio, Casadio *et al*, in this issue).

Remarkably, earlier this year a landscape of cervical carcinomas genomic alterations has revealed previously unknown somatic mutations, suggesting that novel therapeutic strategies and/or biomarkers may soon be considered using these as indicators in the pathogenesis and/or treatment of this disease [45].

## Molecular mechanisms of HPV-mediated carcinogenesis

The genome of all HPV types consists of a circular double stranded DNA filament of about 8 kb, and encodes approximately eight genes. According to protein expression during the viral cycle two functional genome regions have been identified: (i) a coding region containing the early genes, E1, E2, E4, E5, E6, and E7 and (ii) a region containing two late genes, the major (L1) and minor (L2) capsid proteins. In addition, the HPV genome has a non-coding region, termed long control region (LCR), which includes most of the regulatory elements involved in viral DNA replication and transcription [46]. HPV E6 and E7 genes are highly conserved in almost all HPV types so far identified, and in the case of cancer-associated HPV types they encode the major transforming proteins. So far, the majority of the biological studies have been focused on E6 and E7 from HPV16 and HPV18, since they are the most frequent types detected in cervical cancer worldwide.

The majority of sexually active women are infected by mucosal HPV types during their lifetime. Most of these infections remain asymptomatic and are eliminated by the immune system in 6–18 months. Only in a minority of women, HPV infection persists and after a period of latency evolve into low and/or high-grade CINs, which may still regress or progress to an invasive cervical carcinoma [47]. Notably high expression level of E6 and E7 oncoproteins is a biological hallmark of HPV-associated cancers. Another recurrent event occurring during progression to cervical cancer is the transition from episomal to host genome integrated form, even though a subset of HPV16-positive invasive cervical carcinomas, maintains viral DNA only as episomes [48, 49]. In several epidemiological studies, smoking, sexual behaviours, oral contraceptives, and genetic predisposition have been addressed as additional risk factors in the progression of the HPV-mediated disease [10, 50–53] Furthermore, impairment of the immune surveillance appears to facilitate the establishment of a persistent infection and development of a malignant lesion. Organ transplanted or HIV-positive individuals with an immunocompromised status have a much higher prevalence of single or multiple HPV infection and associated lesions than healthy persons [54, 55].

Biological studies have been mainly focused on HPV16 and HPV18, since they are the most frequent types detected in cervical cancer worldwide. These studies have clearly demonstrated that E5, E6, and E7 are directly involved in promoting cellular transformation and altering pathways related to the immune response, as well as cellular transformation [3, 56] by targeting several cellular proteins. One important example of viral/cellular protein interaction is the formation of the complex between E7 and the cellular proteins, pRb, p107, and p130, referred to as pocket proteins. These proteins play a crucial role in the regulation of cell cycle division. In quiescent cells, they directly bind several transcription factors, including members of the E2F family (E2F1-5), inhibiting their activity. In proliferating cells, cyclin-dependent kinases (CDK) become active leading to the phosphorylation of pRb and consequent release of the active forms of E2Fs. HPV16 E7 protein binds the hypo-phosphorylated form of pRb, promoting its degradation via the ubiquitin-proteasome pathway and the progression of the cells into S phase. Thus, HPV16 E7/pRb interaction mimics the CDK-mediated phosphorylation, rendering the cell independent of any type of control. Recently this mechanism has been further defined [57].

The most characterised activity of HPV16 E6 is its ability to degrade the tumour suppressor protein p53 via the proteasome pathway. The p53 is a transcription factor that is activated in response to stress or DNA damage and positively regulates the expression of genes involved in the control of cell cycle arrest or apoptosis. E6 interacts with a 100 kDa cellular protein, E6AP (E6 associated protein), which functions as an ubiquitin protein ligase (E3). The E6/E6AP complex then binds p53, which becomes very rapidly ubiquitinated and, as a consequence, is targeted to proteasomes for degradation. Since the major role of p53 is to safeguard the integrity of the genome by inducing cell cycle arrest or apoptosis, cells expressing HPV16 E6 show chromosomal instability, which greatly increases the probability that HPV-infected cells will evolve towards malignancy. So far, a vast number of E6 and E7 cellular targets have been identified [57, 58]. Many of these cellular proteins are involved in the control of fundamental events, such as proliferation, senescence, apoptosis, differentiation, and immune response. Similarly to p53 and pRb, the majority of the E6 and E7 interactions with the cellular targets result in the degradation of the latter, e.g. the pro-apoptotic protein Bak and NFX1-91, the negative transcriptional regulator of hTERT (human telomerase reverse transcriptase). Interestingly, comparative analyses between the different HPV types led to the identification of E6 and E7 properties that are specific for the high-risk HPV [3]. Because of the complexity and the broadness of the topic, the biology of HPV proteins will not be deeply described in this article. We encourage the readers to consult reviews on the molecular mechanisms of HPV proteins [59, 60]. However, a synthetic summary of some of the biological properties of HPV early protein is given in Table 2.

## Human papillomavirus vaccines

Since 2006 two prophylactic HPV vaccines, Gardasil and Cervarix, are commercially available. They are based on virus-like particles (VLPs) assembled from recombinant main capsid protein L1 produced in eukaryotic systems (Table 3). Both vaccines contain VLPs from HPV16 and HPV18, which, as described above, are responsible for approximately 70% of the cervical cancers worldwide. In addition, Gardasil includes VLPs from the low-risk HPV 6 and 11, which are associated with approximately 90% of the genital warts. The two vaccines also differ in the adjuvant composition; Gardasil is formulated with aluminium as adjuvants, while Cervarix contains additionally the monophosphoryl lipid A (MPL) (Table 3).

Several clinical trials have evaluated the safety and effectiveness of these two products during a follow-up period of seven to eight years. A recent clinical study aiming at directly comparing the two vaccines has clearly demonstrated that so far both products showed high efficacy in preventing the development of cervical pre-malignant lesions, inducing a robust response in immunised individuals and providing protection for at least five years [54, 61, 62].

Although the introduction into the market of this first generation of HPV vaccines represents an important clinical goal in cancer prevention, several additional aspects need to be solved or improved to optimise vaccine efficacy.

HPV-types specificity represents one of the major limitations. Indeed, vaccine efficacy is addressed only to the selected HPV types without any remarkable cross-protection against additional oncogenic HPV types. Hence, both Gardasil and Cervarix do not cover 20–30% of HPV associated cancers. Another important aspect is the high cost of these vaccines, which prevent their widespread application in the vast majority of human population from low and middle resources where cervical cancer is highly prevalent. Modifications of the VLPs assembly chain based on less expensive packaging protocols are currently under evaluation to reduce production costs. In addition, ongoing studies are currently focused on further improving vaccine immunogenicity testing novel antigenic components, such as adjuvants and immune system boosters. This will contribute to obtain a longer-life protection from viral infection and to reduce the number of shots needed for a complete protection [63]. A recent study showed the efficacy of a nonavalent HPV vaccine covering the infection of HPV6, 11, 16, 18, 31, 33, 45, 52 and 58 [60].

Both L1 vaccines have no therapeutic effect in already infected women. Since in the majority of pre-malignant and malignant cervical lesions, E6 and E7 are the only HPV genes expressed, most therapeutic vaccines are focused on the stimulation of an immune-response against these two viral proteins [64]. Recently, very promising results were obtained, using an overlapping peptide pool covering the entire HPV16 E6 and E7 sequences in patients with high-grade vulvar intraepithelial neoplasia [65]. However, additional investigations are required to fully assess the efficacy of HPV therapeutic vaccines.

## Conclusions

The demonstration by epidemiological and biological studies that high-risk HPV infection is associated with human carcinogenesis represents a very important milestone in cancer research. The establishment of such an association led to the development of efficient preventive strategies that has had, and will continue to have a profound impact on public health and overall wealth by reducing the incidence of pre-malignant and malignant cervical lesions. In addition, biological studies on HPV proteins did not only clarify the molecular mechanisms of HPV proteins, but also contributed to our understanding of the fundamental cellular pathways involved in the life of a normal cell.

## Figures and Tables

**Table 1.** Mucosal HPV types (genus alpha) and main associated diseases.

	HPV type	Disease (% attributed cases)
mucosal high-risk	HPV16	Cervical squamous cell carcinoma (~50) Cervical adenocarcinoma (~35) Oropharyngeal cancer (~25)
	HPV18	Cervical squamous cell carcinoma (~20) Cervical adenocarcinoma (~35) Oropharyngeal cancer (~1-3)
mucosal low-risk	HPV31, 33, 35, 39, 45, 51, 52, 56, 58, 59	Cervical squamous cell carcinoma (~30) Minority of oropharyngeal cancers
	HPV6, 11	Benign genital lesions Respiratory papillomatosis
	HPV13, 32	Oral focal epithelial hyperplasia

**Table 2.** Biological properties of HPV Early proteins and their cellular targets.

HPV protein	Functions	Cellular targets
E7	major oncoprotein, cell cycle arrest	pRb, p107, p130
E6	major oncoprotein, cell proliferation, block apoptosis	p53, E6AP, CBP, p300, Bak, hTERT, MAGUK, cIAP, survivin
E5	mediates mitogenic signal of growth factors/early stage transformation stage	—
E4	destabilization of cytokeratin network	—
E2	viral DNA replication and transcription	—
E1	viral DNA replication	—

**Table 3.** Principal features of HPV prophylactic vaccines.

Vaccine commercial name	HPV Types	Adjuvants	Production	Immunological response
Gardasil-Merck	16, 18, 6, 11	Aluminium	Insect cells	Th1
Cervarix-GSK	16, 18	Monophosphoryl lipid A	Yeast	Th1/Th2
